# Prevalence of chronic obstructive pulmonary disease and chronic bronchitis in eight countries: a systematic review and meta-analysis

**DOI:** 10.2471/BLT.21.286870

**Published:** 2022-01-19

**Authors:** Prashant Jarhyan, Anastasia Hutchinson, Damien Khaw, Dorairaj Prabhakaran, Sailesh Mohan

**Affiliations:** a Centre for Chronic Conditions and Injuries, Public Health Foundation of India, Plot No. 47, Sector 44, Gurugram 122002, India.; b Faculty of Health, Deakin University, Burwood, Australia.

## Abstract

**Objective:**

To estimate the prevalence of chronic obstructive pulmonary disease (COPD) and chronic bronchitis in eight countries in South Asia through a systematic review and meta-analysis.

**Methods:**

We searched MEDLINE® Complete, Web of Science, Embase®, Scopus, CINAHL and reference lists of screened studies for research on the prevalence of COPD and chronic bronchitis in South Asian countries published between January 1990 and February 2021. We used standardized diagnostic criteria for definitions of COPD and chronic bronchitis. Two reviewers undertook study screening, full-text review, quality appraisal and data extraction.

**Findings:**

Of 1529 studies retrieved, 43 met the inclusion criteria: 32 provided data from India; four from Bangladesh; three from Nepal; two from Pakistan; and two from both India and Sri Lanka. Twenty-six studies used standardized diagnostic definitions and 19 were included in the meta-analysis. The estimated pooled prevalence of COPD was 11.1% (95% confidence interval, CI: 7.4–14.8%), using the Global Initiative for Chronic Obstructive Lung Disease fixed criteria and 8.0% (95% CI: 5.6–10.4%) using the lower limit of normal criteria. The prevalence of COPD was highest in north India (19.4%) and Bangladesh (13.5%) and in men. The estimated pooled prevalence of chronic bronchitis was 5.0% (95% CI: 4.1–6.0%) in India and 3.6% (95% CI: 3.1–4.0%) in Pakistan.

**Conclusion:**

Included countries have a high prevalence of COPD although it varied by geographical area and study characteristics. Future research in South Asia should use standardized diagnostic criteria to examine the contribution of setting-specific risk factors to inform prevention and control strategies.

## Résumé

**Objectif:**

Estimer la prévalence de la bronchopneumopathie chronique obstructive (BPCO) et de la bronchite chronique dans huit pays d'Asie du Sud par le biais d'une revue systématique et d'une méta-analyse.

**Méthodes:**

Nous avons exploré les bases de données MEDLINE® Complete, Web of Science, Embase®, Scopus et CINAHL, ainsi que des listes de référence d'études sélectionnées, afin de compiler les recherches sur la prévalence de la BPCO et de la bronchite chronique dans les pays d'Asie du Sud publiées entre janvier 1990 et février 2021. Nous avons défini la BPCO et la bronchite chronique en nous fondant sur des critères de diagnostic normalisés. Enfin, deux réviseurs ont trié les études, examiné le texte intégral, évalué la qualité et extrait les données.

**Résultats:**

Sur 1529 études récupérées, 43 correspondaient aux critères d'inclusion: 32 fournissaient des informations sur l'Inde; quatre sur le Bangladesh; trois sur le Népal; deux sur le Pakistan et deux sur l’Inde et le Sri Lanka. Au total, 26 études reposaient sur une définition standard du diagnostic, et 19 étaient intégrées dans la méta-analyse. La prévalence groupée de la BPCO a été estimée à 11,1% (intervalle de confiance de 95%, IC: 7,4–14,8%) selon les critères fixés par l'Initiative mondiale contre la bronchopneumopathie chronique obstructive, et à 8,0% (IC de 95%: 5.6–10.4%) selon la limite inférieure des critères normaux. C'est dans le nord de l'Inde (19,4%) et au Bangladesh (13,5%) que la prévalence la plus élevée a été observée pour la BPCO, et surtout chez les hommes. De son côté, la prévalence groupée de la bronchite chronique a été estimée à 5,0% (IC de 95%: 4,1–6,0%) en Inde et à 3,6% (IC de 95%: 3,1–4,0%) au Pakistan.

**Conclusion:**

Les pays pris en considération présentent une forte prévalence de la BPCO, bien qu'il existe des variations en fonction des zones géographiques et des caractéristiques des études. Les futures recherches menées en Asie du Sud devraient utiliser des critères de diagnostic normalisés pour examiner le rôle des facteurs de risque propres au contexte, afin d'orienter les stratégies de prévention et de lutte.

## Resumen

**Objetivo:**

Estimar la prevalencia de las enfermedades pulmonares obstructivas crónicas (EPOC) y de la bronquitis crónica en ocho países del sur de Asia mediante una revisión sistemática y un metanálisis.

**Métodos:**

Se realizaron búsquedas en MEDLINE® Complete, Web of Science, Embase®, Scopus, CINAHL y en las listas de referencias de los estudios seleccionados en busca de investigaciones sobre la prevalencia de las EPOC y de la bronquitis crónica en los países del sur de Asia publicadas entre enero de 1990 y febrero de 2021. Se utilizaron criterios de diagnóstico estandarizados para las definiciones de EPOC y bronquitis crónica. Dos revisores realizaron la selección de los estudios, la revisión del texto completo, la valoración de la calidad y la extracción de los datos.

**Resultados:**

De los 1529 estudios recuperados, 43 cumplían los criterios de inclusión: 32 proporcionaron datos de la India; cuatro de Bangladesh; tres de Nepal; dos de Pakistán; y dos tanto de la India como de Sri Lanka. Veintiséis estudios utilizaron definiciones diagnósticas estandarizadas y 19 se incluyeron en el metanálisis. La prevalencia conjunta estimada de las EPOC fue del 11,1 % (intervalo de confianza del 95 %, IC: 7,4-14,8 %), utilizando los criterios fijos de la Iniciativa Mundial para las Enfermedades Pulmonares Obstructivas Crónicas y del 8,0 % (IC del 95 %: 5,6-10,4 %) utilizando los criterios del límite inferior de la normalidad. La prevalencia de las EPOC fue mayor en el norte de la India (19,4 %) y en Bangladesh (13,5 %) y en los varones. La prevalencia conjunta estimada de bronquitis crónica fue del 5,0 % (IC del 95 %: 4,1-6,0 %) en la India y del 3,6 % (IC del 95 %: 3,1-4,0 %) en Pakistán.

**Conclusión:**

Los países incluidos tienen una alta prevalencia de EPOC, aunque esta varía según la zona geográfica y las características del estudio. Las investigaciones futuras en el sur de Asia deberían utilizar criterios de diagnóstico estandarizados para analizar la contribución de los factores de riesgo específicos del entorno con el fin de fundamentar las estrategias de prevención y control.

## ملخص

**الغرض:**

تقدير مدى انتشار مرض الانسداد الرئوي المزمن (COPD)، والتهاب الشعب الهوائية المزمن في جنوب آسيا من خلال مراجعة منهجية وتحليل تلوي.

**الطريقة:**

قمنا بالبحث في MEDLINE® Complete، وWeb of Science، وEmbase®‎، وScopus، وCINAHL، وقوائم مرجعية للدراسات التي تم فحصها بغرض الأبحاث عن انتشار مرض الانسداد الرئوي المزمن (COPD)، والتهاب الشعب الهوائية المزمن في دول جنوب آسيا، والتي تم نشرها في الفترة ما بين يناير/كانون ثاني 1990 وفبراير/شباط 2021. كما قمنا باستخدام معايير التشخيص القياسية لتعريفات مرض الانسداد الرئوي المزمن (COPD)، والتهاب الشعب الهوائية المزمن. تولى اثنان من المراجعين فحص الدراسة، ومراجعة النص الكامل، وتقييم الجودة، واستخراج البيانات.

**النتائج:**

من بين الـ 1529 دراسة تم استرجاعها، استوفت 43 منها معايير الاشتمال: قدمت 32 منها بيانات من الهند؛ وأربعة من بنغلاديش؛ وثلاثة من نيبال؛ واثنتان من باكستان؛ واثنتان من كل من الهند وسري لانكا. استخدمت ستة وعشرون دراسة تعريفات تشخيصية قياسية، وتم تضمين 19 منها في التحليل التلوي. كان معدل الانتشار المجمّع التقديري لمرض الانسداد الرئوي المزمن (COPD) هو 11.1% (بفاصل ثقة مقداره: 7.4 إلى 14.8%)، باستخدام المعايير الثابتة للمبادرة العالمية لمرض الانسداد الرئوي المزمن، و8.0% (بفاصل ثقة 95%: 5.6 إلى 10.4%) باستخدام حد أقل للمعايير العادية. كان معدل انتشار مرض الانسداد الرئوي المزمن (COPD) هو الأعلى في شمال الهند (19.4%) وبنغلاديش (13.5%) وفي الرجال. كان معدل الانتشار المجمّع التقديري لمرض التهاب الشعب الهوائية المزمن هو 5.0% (بفاصل ثقة مقداره 95%: 4.1 إلى 6.0%) في الهند و3.6% (بفاصل ثقة مقداره 95%: 3.1 إلى 4.0%) في باكستان.

**الاستنتاج:**

كان لدى البلدان المشمولة نسبة عالية من معدل انتشار مرض الانسداد الرئوي المزمن (COPD) على الرغم من أنها تختلف حسب المنطقة الجغرافية وخصائص الدراسة. يجب أن تستخدم الأبحاث المستقبلية في جنوب آسيا معايير تشخيصية قياسية لفحص مساهمة عوامل الخطر ذات الأوضاع المحددة لزيادة التوعية باستراتيجيات الوقاية والسيطرة.

## 摘要

**目的:**

旨在通过系统评价和元分析来评估南亚八个国家的慢性阻塞性肺疾病 (COPD) 和慢性支气管炎的患病率。

**方法:**

我们搜索了 MEDLINE® Complete、科学网、Embase®、Scopus、CINAHL 和 1990 年 1 月至 2021 年 2 月期间发布的筛查研究参考文献列表来调查南亚国家的 COPD 和慢性支气管炎的患病率。我们采用规范化的诊断标准来确定 COPD 和慢性支气管炎的定义。由两名评审人员负责研究筛选、全文评审、质量评估和数据提取工作。

**结果:**

在检索到的 1529 项研究中，有 43 项符合纳入标准：其中 32 项数据来自印度；4 项来自孟加拉国；3 项来自尼泊尔；2 项来自巴基斯坦；其余两项分别来自印度和斯里兰卡。二十六项研究采用规范化诊断定义，19 项被纳入元分析中。我们采用《慢性阻塞性肺疾病全球倡议》修订版标准评估的 COPD 汇总患病率是 11.1%（95% 置信区间，CI：7.4–14.8%），采用正常标准的下限评估的患病率是 8.0% (95% CI: 5.6–10.4%)。COPD 在北印度 (19.4%) 和孟加拉国 (13.5%) 以及男性间的患病率最高。印度的 COPD 汇总患病率估计值是 5.0% (95% CI: 4.1–6.0%)，巴基斯坦的估计值是 3.6% (95% CI: 3.1–4.0%)。

**结论:**

尽管患病率因地理区域和研究特征而异，但研究所纳入国家的 COPD 患病率较高。南亚地区未来的研究应该采用规范化的诊断标准来检验特定环境的风险因素影响程度，为制定预防和管控策略提供理论依据。

## Резюме

**Цель:**

Оценить распространенность хронической обструктивной болезни легких (ХОБЛ) и хронического бронхита в восьми странах Южной Азии посредством систематического обзора и метаанализа.

**Методы:**

Авторы провели поиск по базам данных MEDLINE® Complete, Web of Science, Embase®, Scopus, CINAHL и по спискам литературы отобранных исследований, опубликованных в период с января 1990 года по февраль 2021 года, для определения распространенности ХОБЛ и хронического бронхита в странах Южной Азии. Для определения ХОБЛ и хронического бронхита авторы использовали стандартизированные диагностические критерии. Два рецензента провели скрининг исследования, обзор полного текста, оценку качества и выборку данных.

**Результаты:**

Из 1529 полученных исследований 43 соответствовали критериям включения: 32 исследования предоставили данные из Индии; четыре — из Бангладеш; три — из Непала; два — из Пакистана и два — из Индии и Шри-Ланки вместе.. В 26 исследованиях использовались стандартизированные диагностические определения, а 19 исследований были включены в метаанализ. Показатель объединенной распространенности ХОБЛ составил 11,1% (95%-й доверительный интервал, ДИ: 7,4–14,8%) при использовании фиксированных критериев Глобальной инициативы по хронической обструктивной болезни легких, и 8,0% (95%-й ДИ: 5,6–10,4%) при использовании нижней границы нормы критериев. Распространенность ХОБЛ была самой высокой среди мужчин в северной Индии (19,4%) и Бангладеш (13,5%). Показатель объединенной распространенности хронического бронхита составил 5,0% (95%-й ДИ: 4,1–6,0%) в Индии и 3,6% (95%-й ДИ: 3,1–4,0%) в Пакистане.

**Вывод:**

Включенные страны имеют высокую распространенность ХОБЛ, хотя она варьировалась в зависимости от географического региона и характеристик исследования. Чтобы разработать стратегии по профилактике и борьбе с заболеванием, в будущих исследованиях в Южной Азии следует использовать стандартизированные диагностические критерии для изучения вклада факторов риска, зависимых от условий.

## Introduction

Chronic obstructive pulmonary disease (COPD) is a common, preventable and treatable disease, with a worldwide prevalence of 10.1% in people aged  40 years or older.^1^^,^^2^ In 2019, COPD was the third leading cause of deaths globally, contributing to 3.23 million deaths, with most deaths (80%) occurring in low-and middle-income countries.^3^^,^^4^ A systematic review on COPD showed that estimates of the number of cases of COPD in countries of the World Health Organization South-East Asia Region had increased from 44.5 million to 75.1 million between 1990 and 2010, a 68.8% increase.^5^

According to the World Bank, South Asia comprises Afghanistan, Bangladesh, Bhutan, India, Maldives, Nepal, Pakistan and Sri Lanka, and is home to a quarter of the global population.^6^ The area is currently undergoing a demographic transition, because of ageing and increased life expectancy.^7^ According to the 2017 Global Burden of Disease study, despite a lower prevalence of COPD in South Asia, the attributable morbidity and premature mortality due to chronic respiratory diseases was highest in South Asia with COPD being the most common cause of premature deaths among chronic respiratory diseases.^8^ The area is also experiencing a change in the burden of risk factors with ambient air pollution becoming a greater risk due to rapid economic development.^8^ While systematic reviews on the prevalence of COPD from Latin America^9^ and sub-Saharan Africa^10^ have been published previously, literature on current prevalence estimates of COPD and its common risk factors in South Asia is scarce.^11^ Notably, few published data exist on the rural–urban, sex, and within and between country differences in the prevalence of COPD. Relevant and timely information on the prevalence of COPD in the area is crucial to inform, develop and implement context-appropriate policies and programmes for its prevention and control, in a setting where the burden is rising.^8^

In COPD, airflow in and out of the lungs is limited due to chronic inflammation and narrowing of airways, which is a result of repeated and long-term exposure of the respiratory tract to noxious stimuli (tobacco smoke, indoor air pollution and repeated respiratory tract infections during childhood).^4^ COPD presents as chronic bronchitis and emphysema.^4^ However, variation in the underlying pathology and clinical presentation, and overlap with the symptoms of asthma and other chronic lung diseases often pose a challenge to making an accurate diagnosis of COPD.^12^ Moreover, the gold standard diagnosis of COPD, which needs evaluation of lung air volumes using a spirometer, requires skilled personnel and quality assurance measures, which makes obtaining accurate data more challenging, especially in low- and middle-income countries where a skilled workforce is limited.^1^^,^^12^

A 2012 systematic review reported limitations in estimates of the prevalence of COPD in India due to a lack of data, concerns about the quality of studies, inconsistencies in study settings and population characteristics.^11^ The availability of more recently published studies provides an opportunity to conduct a systematic review to obtain up-to-date estimates of the prevalence of COPD. These data can be used to inform policy-makers when planning and implementing population-level risk mitigation strategies to control the rising burden of COPD in South Asia.

## Methods

### Design

We conducted a systematic review and meta-analysis of peer-reviewed literature to estimate the prevalence of COPD and chronic bronchitis in South Asia, according to the Preferred Reporting Items for Systematic Reviews and Meta-Analyses guidelines.^13^ We used the World Bank’s classification of countries of the South Asia area: Afghanistan, Bhutan, Bangladesh, India, Maldives, Nepal, Pakistan and Sri Lanka.^6^

We used standardized diagnostic criteria for definitions of COPD and chronic bronchitis. We defined COPD as the presence of persistent airflow limitation according to the Global Initiative for Chronic Obstructive Lung Disease, i.e. a post-bronchodilator ratio of forced expiratory volume in one second (FEV1) to the forced vital capacity (FVC), FEV1/FVC < 0.70 (fixed criteria),^1^ or post-bronchodilator FEV1/FVC below the lower limit of normal, i.e. the lower fifth centile of values from a reference population.^14^ We defined chronic bronchitis as the presence of chronic cough and phlegm according to the criteria of the Medical Research Council in the United Kingdom of Great Britain and Northern Ireland, i.e. cough and sputum production on most days continuously for 3 months for more than 2 consecutive years.^15^

We registered the protocol for this systematic review in the international prospective register of systematic reviews (CRD42020206189).

### Data sources and searches

We searched MEDLINE® Complete, Web of Science, Embase®, Scopus and CINAHL databases using keywords: chronic obstructive pulmonary disease; COPD; obstructive airway disease; obstructive lung disease; airflow obstruction; chronic bronchitis; emphysema; prevalence; and other related terms. These keywords were combined with terms for individual countries and demonyms: Afghan*; Bangladesh*; Bhutan*; India*; Maldiv*; Nepal*; Pakistan*; Sri Lanka*; and associated global areas – South Asia*; Central Asia* (see data repository for search terms).^16^

The study inclusion criteria were (i) community-based studies reporting the prevalence of COPD or chronic bronchitis; (ii) cross-sectional, cohort and case–control design; and (iii) publication date between 1 January 1990 and 28 February 2021. We had no language restrictions. We hand-searched the reference lists of screened studies for additional relevant citations.

### Study selection

We used Covidence for management of systematic review citations.^17^ Two authors independently screened the study titles and abstracts using the inclusion criteria to identify studies for full-text review. These authors undertook an independent full-text review of shortlisted articles, made the final decision to include or exclude studies from the review, independently assessed the methodological quality of included studies using the Joanna Briggs Institute checklist for prevalence studies,^18^ and extracted data from all included studies into a structured data extraction sheet. The following data were extracted from the studies: authors’ names; year of publication and data collection; study title and area; method of disease ascertainment; sample size; sampling technique; study participants; residence; sex; age group; prevalence of COPD and/or chronic bronchitis with their 95% confidence intervals (CIs); and associations between risk factors for and prevalence of COPD and/or chronic bronchitis.

We resolved inconsistencies between reviewers in screening, inclusion and exclusion of studies, quality appraisal and data extraction decisions through discussion. In cases of disagreement, the third reviewer made the final decision. Where necessary, we also contacted the authors of some publications for further information about the methods and data.

### Data analysis

We included studies that used probability sampling techniques and standard definitions for disease ascertainment for the meta-analyses. We did not include studies conducted before 2000 in the meta-analyses. If more than one published study reported the prevalence from the same data set, we included the prevalence from the most recently published study. However, if the older studies provided more details on prevalence data compared with recently published studies, we included the older study data. In the qualitative summary we described characteristics of the studies and charted patterns of the prevalence data according to area, sex and criteria for COPD diagnosis.

We used Stata, version 16.1 (Stata Corp, College Station, United States of America) for all meta-analyses. We used the Stata metaprop command to estimate the pooled prevalence and generate forest plots of COPD (total, men and women) according to fixed criteria and lower limit of normal criteria.^19^ We assessed statistical heterogeneity using the *χ^2^* test, percentage of variance due to heterogeneity using the *I^2^* test and estimated standard deviation of prevalence using the *τ^2^* test. Due to the considerable heterogeneity across studies, we used random-effects models to calculate the pooled prevalence estimates.

## Results

### Included studies

**Fig. 1:**
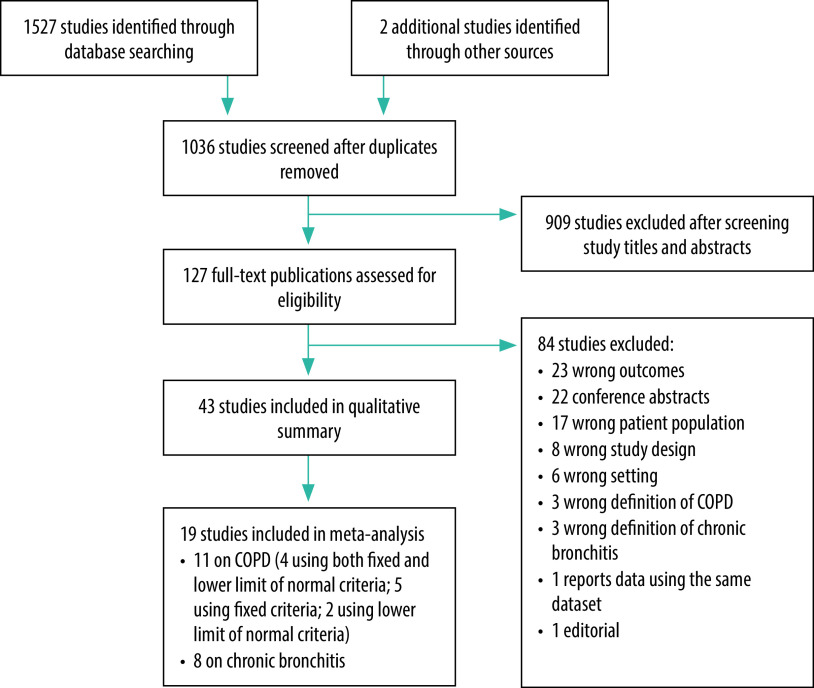
Study selection for the systematic review of chronic obstructive pulmonary disease, eight countries, 2021

Our search of electronic databases and reference lists yielded a total of 1529 studies. After removing 493 duplicates, we screened 1036 titles and abstracts for inclusion and reviewed the full texts of 127 studies. Of these studies, 43 met the inclusion criteria and were included in the qualitative summary and 19 were included in the meta-analysis: 11 studies on COPD with the most recent or detailed data on prevalence of COPD and eight on chronic bronchitis that reported the overall prevalence (Fig. 1).

COPD: chronic obstructive pulmonary disease.

Note: We searched for peer-reviewed literature from Afghanistan, Bhutan, Bangladesh, India, Maldives, Nepal, Pakistan and Sri Lanka.

Of the studies included, 32 were undertaken in India, four in Bangladesh, three in Nepal, two in Pakistan and two in both India and Sri Lanka. Five of the studies were part of the multinational Burden of Obstructive Lung Disease study.^20^^–^^24^ No studies from Afghanistan, Bhutan and Maldives met the inclusion criteria. We judged 16 of the included studies had acceptable quality in all appraisal criteria (data repository).^16^^,^^20^^–^^35^ Only three studies (two from India and one from Nepal) collected the data on COPD prevalence in the past 5 years (Table 1).

**Table 1:** Sample characteristics and study outcomes of research reporting the prevalence of chronic obstructive pulmonary disease, Bangladesh, India, Nepal, Sri Lanka, 2021

Country and study (year data collected)	Study area	Age, years	Sex	Sample size	COPD criteria		Prevalence of COPD based on GOLD fixed criteria, % (95% CI)			Prevalence of COPD based on the lower limit of normal criteria, %		
					Fixed^a^	Lower limit of normal^b^	Overall	Male	Female	Overall	Male	Female
**COPD confirmed with post-bronchodilation spirometry **												
**Studies included in meta-analysis**												
**Bangladesh**												
Alam et al., 2015 (2011–2012)^25^	Rural Matlab and suburban Kamlapur	≥ 40	Male, female	3660	Yes	Yes	Total: 13.5 (12.4–14.6), rural: 17.0, urban: 9.9	Total: 22.0	Total: 6.4	Total: 10.3 (9.3–11.3), rural: 12.5, urban: 8.0	Total:16.2	Total: 5.3
Biswas et al., 2016 (2010–2011)^36^	Rural Chittagong	> 40	Female	250	Yes	No	NA	NA	20.4	NA	NA	NA
Islam et al., 2013 (2008)^26^	Urban Dhaka	≥ 35	Male, female	900	Yes	No	11.4	11.7	10.6	NA	NA	NA
**India**												
Burney et al., 2020 (NR)^24^^c^	Mumbai	≥ 40	Male, female	275 males; 165 females	No	Yes	NA	NA	NA	NR	6.2	7.9
	Pune	≥ 40	Male, female	501 males; 341 females	No	Yes	NA	NA	NA	NR	5.8	6.7
	Srinagar	≥ 40	Male, female	411 males; 341 females	No	Yes	NA	NA	NA	NR	17.3	15.5
	Mysore	≥ 40	Male, female	256 males; 345 females	No	Yes	NA	NA	NA	NR	11.3	5.5
Christopher et al., 2020 (2018)^28^	Rural Vellore	≥ 30	Male, female	787	Yes	Yes	4.1 (2.7–5.5)	5.7	2.9	4.6	4.2	4.9
Johnson et al., 2011 (2007)^29^	Rural Tiruvallur	≥ 30	Female^d^	900	Yes	No	NA	NA	2.4 (1.4–3.5)	NA	NA	NA
Koul et al., 2016 (2010–2011)^21^^c^	Rural Srinagar	≥ 40	Male, female	757	Yes	Yes	19.3	23.7	14.5	16.1	17.3	14.8
Mukhmohit et al., 2014 (NR)^37^	Rural Ambala	≥ 35	Female	1027	Yes	No	NA	NA	5.1	NA	NA	NA
Sinha et al., 2017 (2012–2013)^38^	Urban Delhi	≥ 30	Male, female	1203	Yes	No	10.1 (8.5–11.9)	12.2	7.7	NA	NA	NA
Triest et al., 2019 (NR)^22^^c^	Srinagar	≥ 40	Male, female	739	No	Yes	NA	NA	NA	16.4	NR	NR
	Mumbai	≥ 40	Male, female	440	No	Yes	NA	NA	NA	6.8	NR	NR
	Pune	≥ 40	Male, female	843	No	Yes	NA	NA	NA	6.2	NR	NR
**Nepal**												
Adhikari et al., 2020 (2019)^30^	Semi-urban Pokhara	≥ 40	Male, female	1508	Yes	Yes	8.5 (7.2–10.0)	10.9 (8.7–13.5)	6.4 (4.9–8.4)	5.4 (4.2–6.6)	7.6 (5.8–9.9)	3.5 (2.4–5.0)
**Sri Lanka**												
Triest et al., 2019 (NR)^22^^c^	Colombo	≥ 40	Male, female	1020	No	Yes	NA	NA	NA	7.3	NR	NR
Studies not included in meta-analysis												
Bangladesh												
Grigsby et al., 2016 (2011–2012)^27^^e^	Rural Matlab	≥ 40	Male, female	1846	No	Yes	NA	NA	NA	15.0	NR	NR
	Urban Dhaka	≥ 40	Male, female	1878	No	Yes	NA	NA	NA	10.0	NR	NR
**India**												
Burney et al., 2014^c^: Mumbai (2006–2008); Pune (2008–2009); Srinagar (2010–2011)^20^^e^	Mumbai	≥ 40	Male, female	440	No	Yes	NA	NA	NA	NR	6.0	7.6
	Pune			843	No	Yes	NA	NA	NA	NR	5.7	6.8
	Srinagar			763	No	Yes	NA	NA	NA	NR	17.3	14.8
Townend et al., 2017 (NR)^23^^c,e^	Kashmir	≥ 40	Male, female	738	No	Yes	NA	NA	NA	16.0	NR	NR
Mahesh et al., 2018 (2014–2016)^39^^f^	Rural Mysuru	> 30	Male, female	Phase 1: 8457, phase 2: 1085	Yes	No	0.92	1.0	0.6	NA	NA	NA
**Sri Lanka**												
Townend et al., 2017 (NR) ^23^^c,e^	NR	≥ 40	Male, female	1035	No	Yes	NA	NA	NA	8.0	NR	NR
**COPD without confirmation with post-bronchodilation spirometry**												
**India**												
Arora et al., 2018 (2015)^40^	Urban Delhi	18–59	Female	299^g^	Yes	No	NA	NA	5.0	NA	NA	NA
Chaturvedi et al., 2015 (2014–2015)^41^	Rural Muzaffarnagar	≥ 30	Male, female	908	Yes	No	7.8	NR	NR	NA	NA	NA
Mukherjee et al., 2014 (NR)^42^	Rural West Bengal	23–43	Female^h,i^	1119	Yes	No	NA	NA	2.8	NA	NA	NA
Panigrahi et al., 2018 (NR)^43^	Rural Khordha	18–49	Female^d,i^	1120	Yes	No	NA	NA	All: 22.4 Exposed to biomass fuel smoke: 31.0 Exposed to mixed fuel smoke: 22.8 Not exposed: 7.8	NA	NA	NA
Parasuramalu et al., 2014 (2008)^44^	Rural Bengaluru	> 35	Male, female	1400	Yes	No	4.4	NR	NR	NA	NA	NA
Pathak et al., 2019 (NR)^45^	Rural western Uttar Pradesh	> 18	Female	310	Yes	No	NA	NA	17.42	NA	NA	NA
Shanmugananth et al., 2019 (NR)^46^	Chennai, Surendranagar and Hisar	> 30	Male, female	1000	Yes	No	9.0	NR	NR	NA	NA	NA
Sharma et al., 2016 (2016)^47^	Rural Jammu	> 20	Male, female	2018	Peak expiratory flow rate	No	4.2	5.4	2.8	NA	NA	NA
Sharma et al., 2019 (2012–2013)^48^	Urban Ludhiana	> 20	Male, female	8128	Yes	No	3.2/1000	NR	NR	NA	NA	NA
**Nepal**												
Dhimal et al., 2019 (2016–2018)^49^	Nationwide	≥ 20	Male, female	13 200	Yes	No	11.7 (10.5–12.9)	12.6 (11.2–14.1)	11.0 (9.6–12.4)	NA	NA	NA
Kurmi et al., 2013 (2006–2007)^50^	Rural and urban Kathmandu	≥ 16	Male, female	1392	Yes	Yes	NR	NR	NR	Exposed to biomass fuel smoke: 8.1 Not exposed: 3.6	Exposed to biomass fuel smoke: 7.4 Not exposed: 3.3	Exposed to biomass fuel smoke: 10.8 Not exposed: 3.8

CI: confidence interval; COPD: chronic obstructive pulmonary disease; GOLD: Global Initiative for Chronic Obstructive Lung Disease; NA: not applicable; NR: not reported.

^a^ Fixed criteria defined as a post-bronchodilator ratio of forced expiratory volume in one second (FEV1) to the forced vital capacity (FVC), FEV1/FVC < 0.70.

^b^ The lower limit of normal criteria defined as a post-bronchodilator FEV1/FVC below the lower limit of normal, i.e. the lower fifth centile of values from a reference population.

^c^ Data from the Burden of Obstructive Lung Disease (BOLD) Study.

^d^ Non-smokers.

^e^ Because more recent or detailed data available from another study.

^f^ Only 15% of study participants underwent spirometry.

^g^ 500 women consented and acceptable spirometry data for 299 women were used for analysis.

^h^ Premenopausal women.

^i^ Involved with cooking.

### Prevalence of COPD

Overall, 15 studies reported the prevalence of COPD using the standard diagnostic criteria and post-bronchodilation spirometry (Table 1). Four studies reported data from Bangladesh, eight studies reported data from India, two from both India and Sri Lanka and one from Nepal.^20^^–^^30^^,^^36^^–^^39^ Four studies with data from India^20^^,^^22^^–^^24^ and two from Sri Lanka^22^^,^^23^ reported the overall prevalence of COPD using the lower limit of normal criteria, six studies used the fixed criteria^26^^,^^29^^,^^36^^–^^39^ and four studies (two from India and one each from Bangladesh and Nepal)^21^^,^^25^^,^^28^^,^^30^ used a combination of both the fixed and lower limit of normal criteria (Table 1). Three studies, two from India^29^^,^^37^ and one from Bangladesh,^36^ reported the prevalence using the fixed criteria among women only (Table 1).

**Fig. 2:**
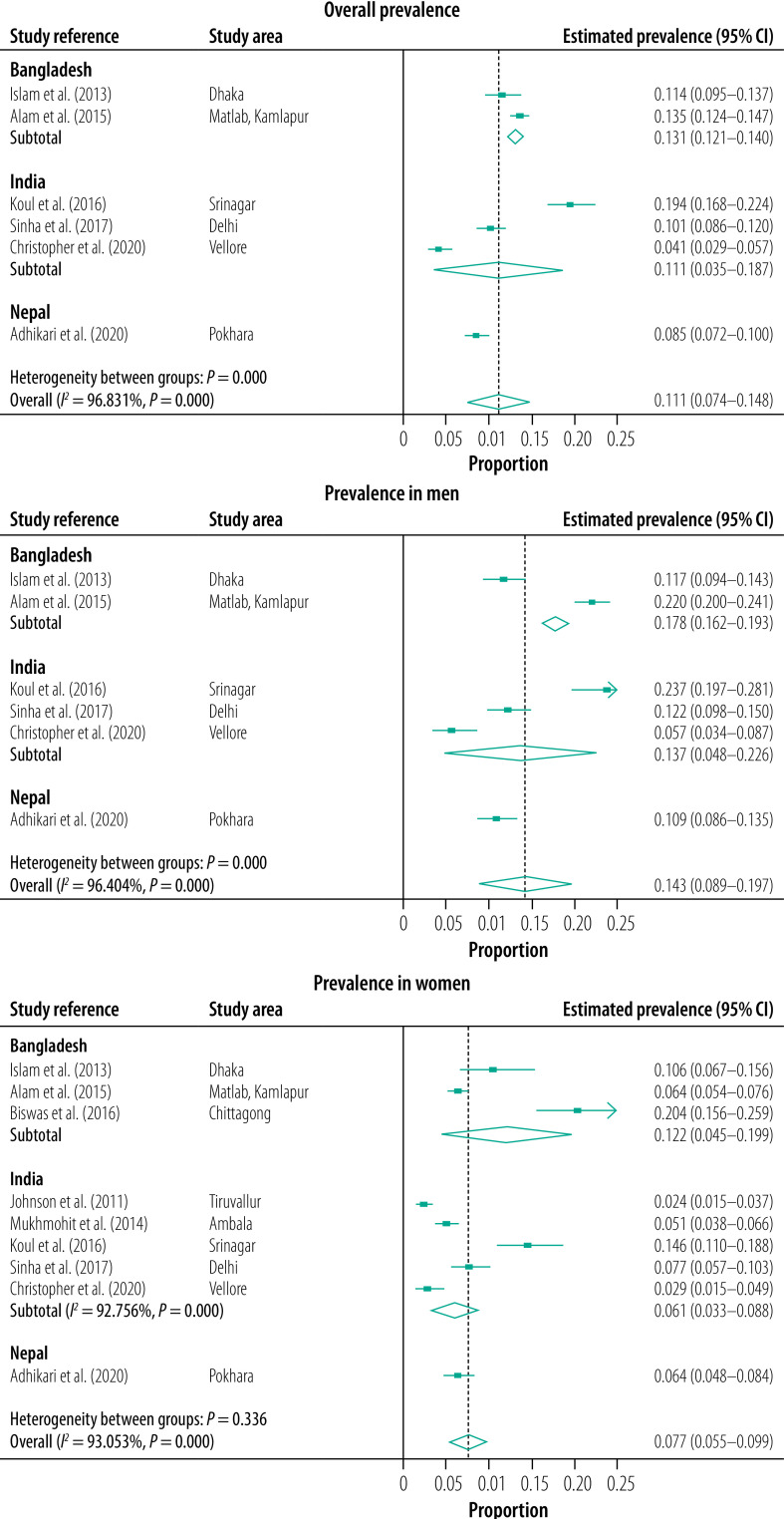
Estimated pooled prevalence of COPD overall and by sex, assessed by the GOLD fixed criteria, Bangladesh, India, Nepal, 2021

COPD: chronic obstructive pulmonary disease; GOLD: Global Initiative for Chronic Obstructive Lung Disease.

Notes: Dashed vertical line represents the pooled effect estimate. For subgroups with three or less studies, statistical analyses were impossible. GOLD fixed criteria is a post-bronchodilator ratio of forced expiratory volume in one second (FEV1) to the forced vital capacity (FVC), FEV1/FVC < 0.70.

**Fig. 3:**
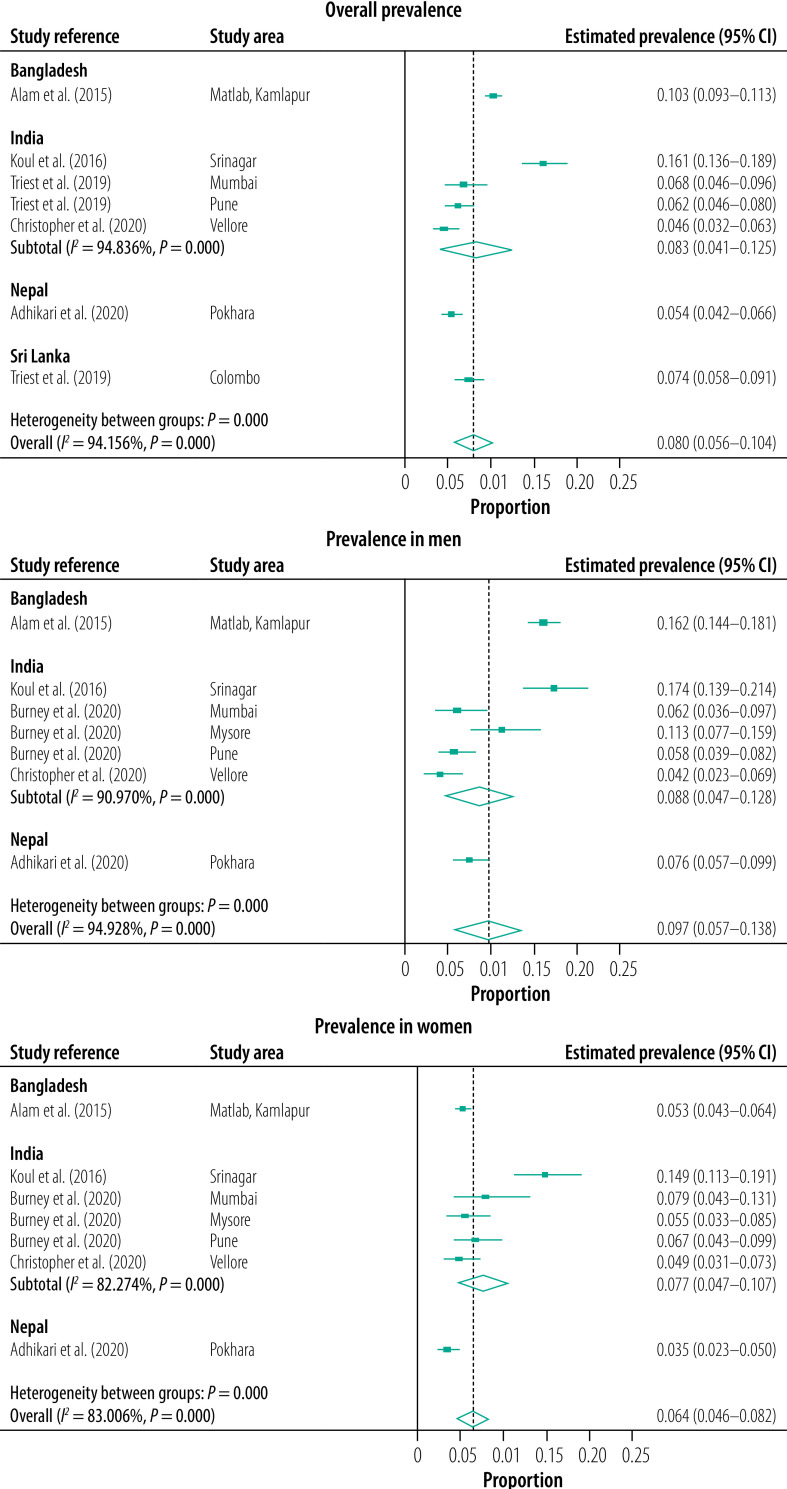
Estimated pooled prevalence of COPD overall and by sex, assessed by the lower limit of normal criteria, Bangladesh, India, Nepal, Sri Lanka, 2021

The estimated pooled prevalence of COPD in the South Asian countries included in our study was 11.1% (95% CI: 7.4–14.8%) using the fixed criteria^21^^,^^25^^,^^26^^,^^28^^,^^30^^,^^38^ (Fig. 2) and 8.0% (95% CI: 5.6–10.4%) using the lower limit of normal criteria (Fig. 3).^21^^,^^22^^,^^25^^,^^28^^,^^30^ The study outcomes had considerable and statistically significant heterogeneity across South Asia (fixed criteria *I^2^*: 96.83%, *P* < 0.001; lower limit of normal criteria *I^2^*: 94.16%, *P* < 0.01), and within India with lower limit of normal criteria *I^2^*: 94.84%, *P* < 0.001.

COPD: chronic obstructive pulmonary disease.

Notes: Dashed vertical line represents the pooled effect estimate. The lower limit of normal criteria is defined as a post-bronchodilator FEV1/FVC below the lower limit of normal, i.e. the lower fifth centile of values from a reference population.

The estimated pooled prevalence of COPD was highest in Bangladesh (13.1%; 95% CI: 12.1–14.0%) followed by India (11.1%; 95% CI: 3.5–18.7) and Nepal (8.5%; 95% CI: 7.2–10.0%; Fig. 2).

In India, the prevalence of COPD varied, with rural Srinagar in northern India having the highest overall prevalence reported in adults aged 40 years or older (19.3% according to the fixed criteria; 16.1% according to the lower limit of normal criteria)^21^ and the lowest prevalence reported in south India (4.1% according to the fixed criteria; Table 1).^28^ A higher prevalence of COPD was also reported in Bangladeshi men from rural Matlab and suburban Kamlapur (22.0% based on fixed criteria; 16.2% based on lower limit of normal criteria; Table 1).^25^

COPD prevalence was found to be significantly higher in men than women in four studies, in Delhi^38^ and Srinagar^21^ according to the fixed criteria, and in Nepal^30^ and rural Matlab, Bangladesh^25^ according to both spirometry criteria. Only one study in Bangladesh compared rural versus non-rural samples and reported a significantly higher prevalence of COPD in rural dwellers (17.0% by fixed criteria and 12.5% by lower limit of normal criteria) than suburban dwellers (9.9% by fixed criteria and 8.0% by lower limit of normal criteria).^25^

### Prevalence of chronic bronchitis

Thirteen studies reported the prevalence of chronic bronchitis and overall prevalence ranged from 2.9% to 9.1% (Table 2).^31^^–^^35^^,^^43^^,^^47^^,^^51^^–^^56^ Studies varied according to residence (three both in rural and urban areas,^32^^,^^33^^,^^51^ nine in rural areas only,^31^^,^^34^^,^^35^^,^^43^^,^^47^^,^^52^^–^^55^ and one was unspecified)^56^ and sex, with eight studies conducted among both men and women,^32^^,^^33^^,^^47^^,^^51^^,^^52^^,^^54^^–^^56^ four studies restricted to women^31^^,^^34^^,^^43^^,^^53^ and one study was restricted to men.^35^ In two multicentre studies in India, the overall prevalence of chronic bronchitis was 4.1% (5.0% in men and 3.2% in women) in 2006^32^ and 3.5% in 2012.^33^ The prevalence of chronic bronchitis was higher in men^32^^,^^47^^,^^51^^,^^54^ and rural dwellers (Table 2).^32^^,^^51^

**Table 2:** Sample characteristics and study outcomes of research reporting the prevalence of chronic bronchitis, India, Pakistan, 2021

Country and study (year data collected)	Study area	Age, years	Sex	Sample size	Prevalence of chronic bronchitis, %				
					Overall	Male	Female	Rural	Urban
**Chronic bronchitis based on standardized criteria included in qualitative summary and/or meta-analysis**									
**India**									
Dutta et al., 2015 (2010–2012)^31^	Rural Wardha (Maharashtra)	≥ 20	Female	1650	NA	NA	2.7	NA	NA
Goel et al., 2007 (2001–2002)^51^	Urban and rural Shimla (Himachal Pradesh)	> 18	Overall, male, female	1330	9.1	11.1	6.1	13.5	4.7
Jindal et al., 2006 (NR)^32^	Urban and rural Chandigarh, Delhi, Kanpur (Uttar Pradesh), Bengaluru (Karnataka)	≥ 35	Overall, male, female	35 295	4.1	5.0	3.2	4.4	3.7 (semi-urban: 6.5)
Jindal et al., 2012 (2007–2009)^33^	Urban and rural: Shimla, Chandigarh, Bikaner, Ahmedabad, Nagpur, Mumbai, Mysore, Trivandrum, Chennai, Secunderabad, Behrampur, Kolkata, Guwahati	≥ 35	Overall	169 575	3.5	NR	NR	NR	NR
Mahesh et al., 2013 (2006–2009)^34^	Rural Mysuru (Karnataka)	> 30	Female	3953	NA	NA	3.4	NA	NA
Mahesh et al., 2014 (2006–2009)^35^	Rural Mysuru	≥ 30	Male	2322	NA	General: 1.7; Smokers: 2.1; Non-smokers: 1.1	NA	NA	NA
	Rural Nanjangud (Karnataka)	≥ 30	Male	2182	NA	General: 21.6; Smokers: 44.8; Non-smokers: 2.0	NA	NA	NA
Panigrahi et al., 2018 (NR)^43^	Rural Khorda (Odisha)	18–49	Female^a,b^	1120	NA	NA	7.3	NA	NA
Sharma et al., 2016 (2012–2013)^47^	Rural Jammu	> 20	Overall, male, female	2018	3.4	4.9	1.7	NA	NA
Spon et al., 2014 (NR)^52^	Rural Kashmir	> 18	Overall, male, female	912	5.4	8.0	3.5	NA	NA
Sukhsohale et al., 2013 (NR)^53^	Rural Nagpur (Maharashtra)	≥ 15	Female^a,b,c^	760	NA	NA	12.5	NA	NA
Viswanathan et al., 2018 (2014–2015)^54^	Rural Kollam (Kerala)	> 15	Overall, male, female	12 556	6.2 (95% CI: 5.8–6.6)	6.7	5.7	NA	NA
**Pakistan**									
Akhtar et al., 2007 (2003–2004)^55^	Rural Peshawar	≥ 10	Overall	2557	5.2	NR	NR	NA	NA
Tageldin et al., 2012 (2010–2011)^56^	Not given	≥ 40	Overall	3654	2.9	NR	NR	NR	NR
**Additional studies on the prevalence of chronic bronchitis**									
**India**									
Akhtar et al., 1999 (NR)^57^	Urban Kashmir	> 30	Overall, male, female	1140	5.7	6.7	4.5	NA	NA
Arora et al., 2018 (2015)^40^	Urban Delhi	18–59	Female	500	NA	NA	NR	NA	NA
Chhabra et al., 2001 (NR)^58^	Urban Delhi	> 18	Male, female^a^	4171	NA	3.1^d,e^	2.1^d,e^	NA	NA
					NA	0.8^d,f^	0.7^d,f^	NA	NA
					NA	1.8^d,g^	0.3^d,g^	NA	NA
					NA	3.2^e,h^	5.9^e,h^	NA	NA
					NA	4.6^f,h^	1.1^f,h^	NA	NA
					NA	0.5^g,h^	1.7^g,h^	NA	NA
Jindal, 1993 (NR)^59^	Urban and rural Chandigarh	NR	Overall, male, female	1475	2.4	2.1	1.6	NR	NR
Mahesh et al., 2009 (NR)^60^	Rural Mysuru (Karnataka)	> 40	Overall, male, female	900	7.1^i^	11.1^i^	4.5^i^	NA	NA
Pandita et al., 2017 (NR)^61^	Urban Dehradun (Uttarakhand)	≥ 60	Male, female	520	25.0^j^	NR	NR	NA	NA
Qureshi, 1994 (NR)^62^	Rural Gandarbal (Kashmir)	> 15	Male, female	560	7.7	NR	NR	NA	NA
Shanmugananth et al., 2019 (NR)^46^	Chennai (Tamil Nadu), Surendranagar (Gujarat), Hisar (Haryana)	> 30	Male, female	1000	4.1^k^	NR	NR	NA	NA

CI: confidence interval; COPD: chronic obstructive pulmonary disease; NA: not applicable; NR: not reported.

^a^ Non-smokers.

^b^ Involved in cooking.

^c^ Not pregnant.

^d^ Low pollution zone.

^e^ Low socioeconomic status.

^f^ Middle socioeconomic status.

^g^ High socioeconomic status.

^h^ High pollution zone.

^i^ Validation study followed by pilot study.

^j^ Conducted among elderly and chronic bronchitis/COPD definitions not mentioned.

^k^ Purposive sampling used.

**Fig. 4:**
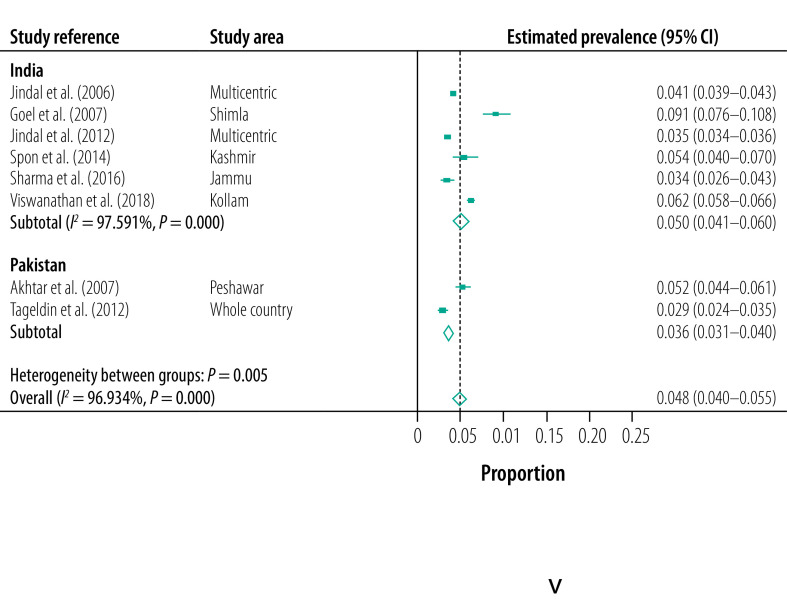
Estimated pooled prevalence of chronic bronchitis, India, Pakistan, 2021

Notes: Dashed vertical line represents the pooled effect estimate. For subgroups with three or less studies, statistical analyses were impossible.

Eight studies (six from India and two from Pakistan), which diagnosed chronic bronchitis using standardized criteria and provided the overall prevalence of chronic bronchitis, were used in the meta-analysis (Table 2).^32^^,^^33^^,^^47^^,^^51^^,^^52^^,^^54^^–^^56^ The overall estimated pooled prevalence of chronic bronchitis was 4.8% (95% CI: 4.0–5.5%): 5.0% (95% CI: 4.1–6.0%) in India and 3.6% (95% CI: 3.1–4.0) in Pakistan (Fig. 4). Statistical heterogeneity was very high and statistically significant for all the studies included (*I^2^*: 96.93%; *P* < 0.001) and for studies undertaken in India (*I^2^*: 97.59%; *P* < 0.01). There was an insufficient number of relevant studies to calculate statistical heterogeneity in Pakistan.

Common risk factors associated with COPD and chronic bronchitis were age, smoking, lower socioeconomic status, exposure to environmental tobacco smoke, exposure to biomass fuel, exposure to dust, history of tuberculosis and history of allergy and/or asthma.^20^^,^^21^^,^^24^^–^^26^^,^^28^^,^^30^^,^^32^^,^^33^^,^^36^^–^^38^^,^^51^^,^^55^^,^^56^

## Discussion

Here we report on the prevalence of COPD and chronic bronchitis in South Asia. A substantial regional variation was seen in the prevalence of COPD and chronic bronchitis, with higher prevalence estimates reported by studies in north India^21^ and Bangladesh.^25^ While tobacco smoking and indoor air pollution were the most common risk factors assessed for their association with COPD, no population-based studies were found in the area that determined the association of COPD with other important risk factors such as ambient air pollution and occupational hazards.

Within-country and between-country variations in the prevalence of COPD have been reported previously due to differences in the prevalence of risk factors, especially tobacco smoking.^63^^,^^64^ The higher prevalence of COPD in north India (Kashmir) was mainly ascribed to tobacco smoking using traditional hookahs and higher exposure to indoor air pollution.^21^ The high prevalence of COPD in Bangladesh was also attributed to the high prevalence of tobacco smoking, particularly among men.^25^ Traditional norms of offering smoking products, low awareness of the harmful effects of smoking among low-income groups and people living in rural areas, suboptimal implementation of tobacco control measures and limited access to cessation services may account for high prevalence estimates in these areas.^65^ Thus, these relevant regional, sociocultural and economic factors need to be considered while planning strategies to reduce smoking, decrease the COPD burden and improve population lung health.^65^

Indoor air pollution is one of the main causes of COPD, especially in South Asian women.^66^ Although many governments have scaled up access to cleaner cooking fuels, the reach and change to cleaner fuels is suboptimal. Furthermore, the effect of this transition on the COPD burden, especially among women in South Asia, remains unclear and warrants further evaluation.^67^ More specific approaches are required to understand the role of various indoor air pollutants, such as particulate matter, nitrogen dioxide, carbon monoxide, sulfur oxides, polycyclic organic matter and formaldehyde which are produced by combustion of biomass fuels, in the development of COPD and chronic bronchitis.^68^^,^^69^

Ambient air pollution is one of the main risk factors for COPD mortality and disability-adjusted life years lost, with the highest burden reported in South Asia.^8^^,^^70^ In 2015, Bangladesh, India and Nepal had the highest burden of particulate matter 2.5 (PM_2.5_) and Bangladesh, India and Pakistan had the highest increase in the ozone levels and the highest mortality due to ambient PM_2.5_ observed.^71^ Increased air pollution aggravates COPD symptoms, known as COPD exacerbations, and increases hospitalizations and mortality.^72^ Although a few studies from large cities in India have shown the short-term effect of increased ambient air pollution and increased hospital visits due to respiratory problems,^73^^,^^74^ the long-term effects of extended exposure to ambient air pollution and its effects on lung function, morbidity and mortality need to be studied.^69^^,^^75^ Educating health-care providers and patients about the adverse health effects of ambient air pollution and simple measures that can be taken to reduce exposure, such as avoiding going out during periods of high pollution and wearing masks outdoors, is needed.^76^

Few studies have assessed the association between COPD and respiratory infections in South Asia, such as lower respiratory tract infections and tuberculosis.^21^^,^^24^^,^^30^ Given that many people in the area have been affected by severe acute respiratory syndrome coronavirus 2 (SARS-CoV-2),^77^ the long-term effects of this virus on the burden of COPD and other chronic respiratory diseases need to be assessed. Poor lung function associated with SARS-COV-2 and respiratory infections also requires monitoring to assess their long-term effects and the attributable risk for the development of COPD.

We also found that none of the included studies had assessed the presence of chronic bronchitis with airway obstruction in COPD. Chronic bronchitis in people with airway obstruction is associated with more severe disease, poor general health status, greater limitations on physical activity and higher mortality.^78^ Therefore, COPD should be assessed together with the presence of chronic bronchitis in both clinical and research settings.

In our study, we tried to assess the current prevalence of COPD in South Asian countries. The strengths of our study include selection of recent studies, a comprehensive literature search using explicit definitions for COPD and chronic bronchitis according to international guidelines and a comprehensive quality assessment of studies selected for the review.

Our study has some limitations. First, because few studies in the area were available, our pooled prevalence estimates of COPD cannot be generalized to all South Asian countries. Only one study each from Bangladesh and India assessed the prevalence of COPD in urban areas and the only study from Nepal assessed the prevalence in a semi-urban area. Moreover, due to within-country differences in the burden of risk factors, especially in India,^79^ the generalizability of the prevalence estimates may be limited to the states or areas where the studies were conducted. There were too few studies to calculate statistical heterogeneity within Bangladesh, Nepal and Sri Lanka, and to identify predictors of heterogeneity using meta-regression. However, visual inspection of the forest plots suggests variations in the prevalence of COPD by country, geographical location and sex. Second, most studies included in the systematic review had collected data more than 5 years ago. In recent times, the burden of risk factors for COPD and chronic bronchitis has changed considerably. For example, smoking rates decreased from 14.0% to 10.7% between 2009–2010 and 2016–2017 in India,^80^ while the ambient air pollution has increased in South Asia.^81^ These limitations highlight the lack of research to determine the accurate burden of COPD in the area and the need for large population-based studies with rigorous methods to generate accurate prevalence estimates in various population subgroups as well as attributable risks of common risk factors, including outdoor air pollution, occupational exposure and infections. Such data are important to inform the development and implementation of context-relevant policies and programmes for reducing the increasing burden of COPD and its risk factors.

Estimation of the prevalence of COPD is challenging for researchers in low- and middle-income countries. As a result, there is likely a large burden of undetected COPD. COPD is a complex disease with varied presentations and new phenotypes being identified.^82^^,^^83^ Conducting post-bronchodilation spirometry, which is required for the confirmation of a diagnosis of COPD, is resource intensive and requires a high level of quality control.^1^^,^^8^ Different spirometry criteria for diagnosing COPD result in within-study differences in the prevalence estimates, which further complicates the interpretation of the disease burden, especially for policy-makers who need to allocate resources for COPD control. This problem emphasizes the need for standardized COPD diagnosing criteria that can be implemented in low- and middle-income countries.

Some studies have suggested screening strategies for early detection of COPD.^84^^,^^85^ However, COPD screening needs further evaluation and feasibility studies and should be supported by strengthening of public health infrastructure for the confirmation of diagnosis, early initiation of pharmacological and non-pharmacological treatment including pulmonary rehabilitation.^85^

In conclusion, given the paucity of studies on the current burden of COPD and its risk factors in most South Asian countries, future research in these countries should ensure that standardized diagnostic criteria are used to examine the contribution of exposure to context-relevant risk factors to inform COPD prevention and control policies.
